# Validation of the multidimensional WHOQOL‐OLD in Ghana: A study among population‐based healthy adults in three ethnically different districts

**DOI:** 10.1002/brb3.2193

**Published:** 2021-06-17

**Authors:** Adote Anum, Samuel Adjorlolo, Charity S. Akotia, Ama de‐Graft Aikins

**Affiliations:** ^1^ Department of Psychology University of Ghana Legon‐Accra Ghana; ^2^ Department of Mental Health School of Nursing and Midwifery College of Health Sciences University of Ghana Legon‐Accra Ghana; ^3^ Research and Grant Institute of Ghana Accra Ghana; ^4^ Department of Psychology University of Ghana Legon‐Accra Ghana; ^5^ Institute of Advanced Studies University College London London UK; ^6^ Regional Institute for Population Studies University of Ghana Legon‐Accra Ghana

**Keywords:** aged, Ghana, quality of life, WHOQOL‐OLD

## Abstract

**Objectives:**

Study of well‐being of older adults, a rapidly growing demographic group in sub‐Saharan Africa, depends on well‐validated tools like the WHOQOL‐OLD. This scale has been tested on different populations with reasonable validity results but has limited application in Africa. The specific goal of this paper was to examine the factor structure of the WHOQOL‐OLD translated into three Ghanaian languages: Ga, Akan, and Kasem. We also tested group invariance for sex and for type of community (distinguished by ethnicity/language).

**Methods:**

We interviewed 353 older adults aged 60 years and above, selected from three ethnically and linguistically different communities. Using a cross‐sectional design, we used purpose and convenience methods to select participants in three geographically and ethnically distinct communities. Each community was made up of selected rural, peri‐urban, and urban communities in Ghana. The questionnaire was translated into three languages and administered to each respondent.

**Results:**

The results showed moderate to high internal consistency coefficient and factorial validity for the scale. Using confirmatory factor analysis, we found that the results supported a multidimensional structure of the WHOQOL‐OLD and that it did not differ for males and females, neither did it differ for different ethnic/linguistic groups.

**Conclusions:**

We conclude that the translated versions of the measure are adequate tools for evaluation of quality of life of older adults among the respective ethnic groups studied in Ghana. These results will also enable comparison of quality of life between older adults in Ghana and in other cultures.

## Graphical abstract

The need for a validated measure is important for cross‐cultural studies of well‐being. In a study among older adults in a sub‐Saharan African country, we found that the WHOQoL‐OLD measure is an adequate tool for evaluation of quality of life. We did not find group invariance suggesting comparability of quality of life across different ethnicities.

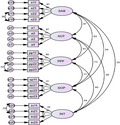

**Keypoints**

1. The WHOQOL_OLD has factorial validity in a cross‐cultural context.
2. Translation into multiple languages does not affect the factor structure.
3. There are no differences in sex on the issue of quality of life.
4. It appears death and dying is an uncomfortable subject among older adults.

## 1 INTRODUCTION

The rapid pace of economic development in low‐ and middle‐income countries (LMICs) has resulted in demographic shifts from younger populations to a more aged population. This is fueled largely by improved health care and increase in life expectancy in many LMICs (Ahmad, 2016; Prina et al., 2020). Improved health of the population in LMICs is accompanied by improved quality of life which also means that more people are living into advanced old age (Ahmad, 2016; Gyasi & Phillips, 2020). The demographic shift in life expectancy has caused changes in the disease burden profiles of LMICs, with chronic noncommunicable diseases (NCDs) becoming a more common and growing public health challenge (Aikins & Agyeman, 2017). Consistent with this change, governments’ concerns are moving toward developing comprehensive policies on provision of interventions that meet the health needs of older adults. An effective comprehensive policy is developed on the basis of accurate need‐based scientific research. There is increasing effort to provide this need‐based research globally, especially on physical health needs of the aged.

In LMICs, especially in sub‐Saharan African countries like Ghana, the research is gradually shifting from physical health needs research to quality of life and mental health research, but this change is slow and therefore limited information is available on psychological health needs of the older adults (Aikins & Apt, 2016). This gap has slowed the ability to generate evidence‐based policies and interventions to meet the psychological health needs of the increasing adult population. The major reason for this is that behavioral and mental health research in LMICs is dogged partly by limited and skewed allocation of funding resources (Anum et al., 2020). One important element for research in quality of life and mental health is the use of contextually validated tools that are accurate and allow for cross‐cultural comparability. Mental health intervention policies for older adults will require extensive investment into accurate assessment and diagnosis of psychological well‐being and/or psychiatric morbidity.

In response to developing cross‐culturally validated measure of well‐being, the World Health Organization Quality of Life Group has developed the quality of life measures, the WHOQOL‐100, and its short form to address the issue of measurement of quality of life (WHOQOL Group, 1993, 1998). Another purpose is to develop a measure that has cross‐cultural relevance. Considering the applicability of these two instruments for older adults, the WHOQOL research group developed a WHOQOL‐OLD module for older adults, containing six facets (Power et al., 2005). This measure has been translated into several language versions with reasonable psychometric properties (Eser et al., 2010; Fleck et al., 2006).

The World Health Organization quality of life instrument for older adults largely assesses multifaceted quality of life and psychological well‐being. The measure has been used to accurately distinguish between depressed patients and patients in remission (Hussenoeder et al., 2020; Skevington et al., (2020)) or healthy patients (Bonicatto et al., 2001). Although the measure provides adequate validity coefficients, there are differences that result from cultural specificities. It was therefore recommended that it is important to validate the tool within each cultural context (Fleck et al., 2006; Power et al., 2005).

We designed the study to examine the validity of the instrument in a multiethnic and multilingual population. We therefore tested the factorial validity of the WHOQOL‐OLD among a cross section of healthy older adults in Ghana. Specifically, (1) we tested the assumption that the underlying dimensions of the measure would be confirmed, and we also examined whether (2) the WHOQOL‐OLD is invariant for males and females and (3) invariant for three ethno‐linguistic groups.

It was our expectation that the use of the WHOQOL‐OLD among Ghanaians will yield a concise evaluation of older people's rating of their quality of life and furthermore provide caregivers, healthcare providers, and potentially policy makers with a more holistic idea of what older people need in order to have quality of life.

## 2 METHODOLOGY

### 2.1 Research setting and sampling

The sample for this study was selected using a multi‐stage process that began with selection of districts and then households. Three districts were purposively selected: Accra (Ga West district) in the south, Sunyani (Sunyani East and West districts) in the middle belt, and Navrongo in the Upper East Region. Three factors guided the selection of the districts. First, the districts are geographically and ethnically distinct, and they have easily accessible rural, peri‐urban, and urban communities. Second, their locations in the South, Middle, and the Northern‐most parts of the country ensured that the sample for the study was close to a nationally representative sample. Third, all three districts had their unique languages which then allowed us to examine the factorial validity of the quality of life scale in ethno‐linguistically different groups. The selected ethnic groups are three of the six major ethnic groups in Ghana. Each selected district has a dominant language although other languages may be spoken within the districts.

The criteria for inclusion were that the participants had to be 60 years and above and did not have any signs of ill‐health that could impede their ability to participate in the interview. In each selected district, a community facilitator who lives in and knows the community very well was hired to help stratify the communities in the districts in order to select participants from all different sections of the selected district. In each selected locality, any household with an individual who was 60 years and older was contacted. The distribution of the sample is presented in Table 1.

### 2.2 Measures

The World Health Organization Quality of Life‐Old (WHOQOL‐OLD) is a 24‐item, 6‐facet instrument with cross‐cultural reliability (Power et al., 2005; Van Biljon et al., 2015). This was developed by the World Health Organization Quality of Life group, a collaborative effort among numerous researchers from various countries which led to the development of a measure focusing on the quality of life in older population cohorts (Power et al., 2005). As indicated, there are six facets or domains, which are Sensory abilities, Autonomy, Past, present, and future, Social interaction, Death and dying, and Intimacy. Each facet is measured by four items. The original version, designed to assess quality of life cross‐culturally in health and health care, the WHOQOL, has 100 items. We also asked questions about health status and other demographic characteristics required for the study.

### 2.3 Translation procedure

The standard translation and back translation methods were used. First, the original WHOQOL‐OLD was translated into the three respective local languages: Ga in Accra, Akan in Sunyani, and Kasem in Navrongo, following WHO translation guidelines for assessment of instruments (Üstun et al., (2005)). Second, the translated versions were back translated into the English language by other language experts who were not familiar with the original English version.

The third step involved the evaluation of the back‐translated versions, comparing them to the original WHOQOL‐OLD by the first author who is literate in Akan and Ga languages. In the case of Kasem, the back‐translated version was evaluated with one of the research assistants. During this phase, the first author corrected any discrepancies, focusing on contextual and linguistic meaning. Items that lacked clarity were referred to the translators. The final phase involved a discussion of contextual and linguistic equivalence of the items during training. The first author who did the training led the discussion on the items, and when there was no consensus, the item(s) was referred to the translators.

### 2.4 Data collection procedure

Eight research assistants were trained for the study; five had degrees in psychology, one had a degree in sociology, one had a degree in social work, and one had a degree in education. The training of research assistants was in two phases. The first phase involved training on the original English version. In the second phase, the research assistants were trained on the translated versions of the questionnaire. During this phase, the research assistants and the first author had discussions about contextual and linguistic accuracy and items on which there was no consensus were referred to the translators.

We pretested the questionnaire in a sample of 25 older adults in a peri‐urban town in the northern part of Accra. This is a typical Ghanaian community which shared similar characteristics with the communities for the main study. There were minimal modifications to item translations following feedback from the participants. For example, one item on the Death and Dying dimension—“Fear pain before death”—was deleted after multiple translations could not result in consensus on meaning of the item.

All participants completed the WHOQOL‐OLD scale and a demographic questionnaire that included questions about age, marital status, living arrangements, occupational, and income statuses. We administered the questionnaire in the dominant language for each district. Interviews were conducted by trained interviewers (research assistants) with the use of the questionnaire as described in measures.

### 2.5 Ethical issues

The research received ethical approval from the Ethical Committee for Humanities of the University of Ghana. The study number is ECH 105/17‐18. The research was done in compliance with ethical requirements in Ghana. All participants signed or thumb‐printed an informed consent form. Participants received either phone credits or cakes of soap valued at five Ghana cedis (approximately $1.00).

### 2.6 Statistical analyses

The questionnaires were coded into SPSS Version 24. The data were managed and analyzed using this software. The Cronbach's alpha coefficients were estimated to assess internal consistency. A series of confirmatory factor analysis (CFA) was also conducted to test for the theorized model and factorial structure and to test for group invariance.

## 3 RESULTS

The descriptive statistics of background characteristics of the study sample are presented in Table 1. The sample is made up of 353 older adults (> 60 years of age) living in rural, peri‐urban, and urban communities in three districts (Ga West, Navrongo, and Sunyani, in Ghana).

Majority of the respondents were females (67.2%), and the average age was 71.65 years. Almost two‐thirds of the respondents were not married or lived with a regular partner. More than 70% were not employed or on a regular income which is expected of the demographic group studied.

**TABLE 1:** Descriptive statistics for key variables in the study participants

Measures (*N*)	Percent	Mean	*SD*	Min	Max
Study site					
Ga West (111)	31.40				
Navrongo (120)	34.00				
Sunyani (122)	34.60				
Age		71.65	9.22	60	85
Sex					
Female (229)	67.20				
Male (112)	32.80				
Marital status					
Married (125)	35.40				
Unmarried (228)	64.60				
Employment status					
Employed (96)	27.30				
Unemployed (257)	72.50				
Income status					
Regular income (105)	29.70				
Nonregular income (248)	70.30				
WHOQOL‐OLD Domains					
Sensory abilities (352)		13.25	4.36	4	20
Autonomy (353)		15.62	3.74	4	20
Past, present, future (351)		15.14	3.61	4	20
Social interaction (351)		12.99	4.69	4	20
Death and dying^a^ (347)		5.03	3.16	3	15
Intimacy (351)		15.75	3.78	4	20

^a^ Based on three items

Internal consistency was measured using the Cronbach alpha coefficient, and they were all found to be reasonably acceptable ranging from 0.844 to 0.937 for the WHOQOL domains. These are considered moderate to high coefficients (Cortina, 1993). Death and dying has the lowest mean score. As indicated earlier, one item, “Fear pain before death,” was not included in the main study because its inclusion resulted in low psychometric coefficients across all the sites. Two other items on this domain—“Afraid of not being able to control Death” and “Scared of dying”—were within acceptable Skewness and Kurtosis limits of 2.0 (George & Mallery, 2019). Intimacy has the highest mean score.

The correlation analysis showed there were moderate correlations among the WHOQOL subdimensions. The correlation with death and dying was the lowest. The results for correlations are presented in Table 3.

**TABLE 2:** Item means, standard deviations, and internal consistency coefficients of the WHOQOL‐OLD items

Item Number	Domain	Description	Mean	*SD*	Skewness	Kurtosis	Cronbach's Alpha
1	*Sensory abilities*	Impairment to senses affect daily life	3.33	1.19	−0.498	−0.736	0.937
20		Rate sensory functioning	3.35	1.18	−0.403	−0.839	
2		Loss of sensory abilities affect participation in activities	3.35	1.18	−0.625	−0.602	
10		Problems with sensory functioning affect ability to interact	3.27	1.16	−0.464	−0.723	
3	*Autonomy*	Freedom to make own decisions	4.16	0.967	−1.536	2.437	0.885
4		Feel in control of your future	3.69	1.044	−0.743	0.184	
11		Able to do things you'd like to do	3.63	1.225	−0.844	−0.253	
5		People around you are respectful of your freedom	4.14	1.086	−1.396	1.305	
19	*Past present future*	Happy with things to look forward to	3.68	1.036	−0.780	0.184	0.844
12		Satisfied with opportunities to continue achieving	3.52	1.077	−0.718	−0.067	
13		Received the recognition you deserve in life	3.97	1.381	−1.070	0.352	
15		Satisfied with what you've achieved in life	3.96	1.304	−1.165	0.720	
16	*Social interaction*	Satisfied with the way you use your time	3.49	1.086	−0.524	−0.348	0.908
17		Satisfied with level of activity	3.52	0.899	−0.470	−0.001	
14		Have enough to do each day	3.61	1.032	−0.695	−0.082	
18		Satisfied with opportunities to participate in the community	3.69	0.981	−0.672	0.002	
6	*Death & dying*	Concerned about the way you will die	2.05	1.382	0.871	−0.797	0.920
7		Afraid of not being able to control Death	1.50	1.084	2.049	2.797	
8		Scared of dying	1.48	1.417	2.260	3.588	
9^a^		Fear pain before death					
21	*Intimacy*	Feel a sense of companionship in life	3.72	0.995	−0.799	0.351	0.930
22		Experience love in your life	3.95	0.904	−1.024	1.217	
23		Opportunities to love	4.06	0.900	−1.134	1.431	
24		Opportunities to be loved	4.02	0.916	−0.997	0.881	

^a^ This item was deleted from the main study because of poor internal consistency indicator during pretesting of the questionnaire.

**TABLE 3:** Correlations (Pearson *r*) among key constructs

	Constructs	2	3	4	5	6	7
1	Sensory abilities	0.621**	0.504**	0.600**	−0.143*	0.354**	0.384**
2	Autonomy		0.635**	0.676**	−0.114*	0.550**	0.519**
3	Past, Present, Future			0.616**	−0.154**	0.557**	0.634**
4	Social Interaction				0.069	0.527**	0.455**
5	Death & Dying					−0.028	0.170**
6	Intimacy						0.422**
7	Overall WHOQOL‐OLD score						

* =0.05.

** =0.001.

### 3.1 Confirmatory factor analysis

Confirmatory factor analysis (CFA) was used to estimate the factor structure of the WHOQOL in the full sample. This was followed by multi‐group CFA to determine measurement invariance of the WHOQOL based on sex and geographical location. In the first of series of analyses, separate models were estimated for males and females, followed by estimation of unconstrained (baseline) model. In this model, the parameters were freely estimated across the groups, with satisfactory fit indices indicating the attainment of a configural invariance. In subsequent model estimations, some constraints were introduced, with each successive model containing all the constraints of its predecessor. First, the factor loadings were held constant across the groups to investigate metric invariance, followed by covariance and variances, and lastly error variances were held to be equal for the groups to determine invariance of the covariance and variances, and error variances, respectively. To determine sex invariance, differences in comparative fit index (CFI; ΔCFI) and chi‐square (χ^2^; Δχ^2^) were used. A nonsignificant Δχ^2^ and ΔCFI ≥ −0.01 between the restrictive and less restrictive or unconstrained models indicate the attainment of sex invariance. Regional invariance determination followed the procedure above.

Model fit was determined using the following common fit indicators: χ^2^, CFI, Tucker–Lewis Index (TLI), and a noncentrality‐based index, the root mean square error of approximation (RMSEA). The CFA and multi‐group CFA were conducted with maximum likelihood estimation method in Analysis of Moment Structures (AMOS) version 21 (Arbuckle, 2011).

Preliminary analysis in the CFA revealed that the model did not provide a good fit to the data (TLI = 0.74; CFI = 0.87; RMSEA = 0.10). Inspection of the items constituting the various dimensions of the scale showed that the items for Death and Dying loaded poorly (i.e., ≤. 15) and were not significant (*p* >.05). A decision was reached to exclude the Death and Dying dimension from further analysis.

### 3.2 Confirmatory factor analysis and sex invariance

The CFA and sex invariance analysis results are summarized in Table 4. The initial model for the full sample did not provide a good model fit to the data (TLI = 0.92; CFI = 0.94; RMSEA = 0.09). Based on the modification indices (MI), the model was respecified by allowing the residuals of the following items to correlate: Intimacy #22 (Experience love in your life) and Past, present and future #15 (Satisfied with what you've achieved in life) (MI = 92.92), Intimacy #11 (Feel a sense of companionship in life) and Intimacy #21 (Experience love in your life ) (MI = 25.09), and Sensory abilities #22 (Rate of sensory functioning) and Sensory abilities #23 (Loss of sensory abilities affect participation in activities) (MI = 15.55). The results showed that respecified model was an improvement over the initial model (Δχ^2^ = 158.25, *p* < .001), providing a good model fit (TLI = 0.95; CFI = 0.96; RMSEA = 0.06).

**FIGURE 1:**
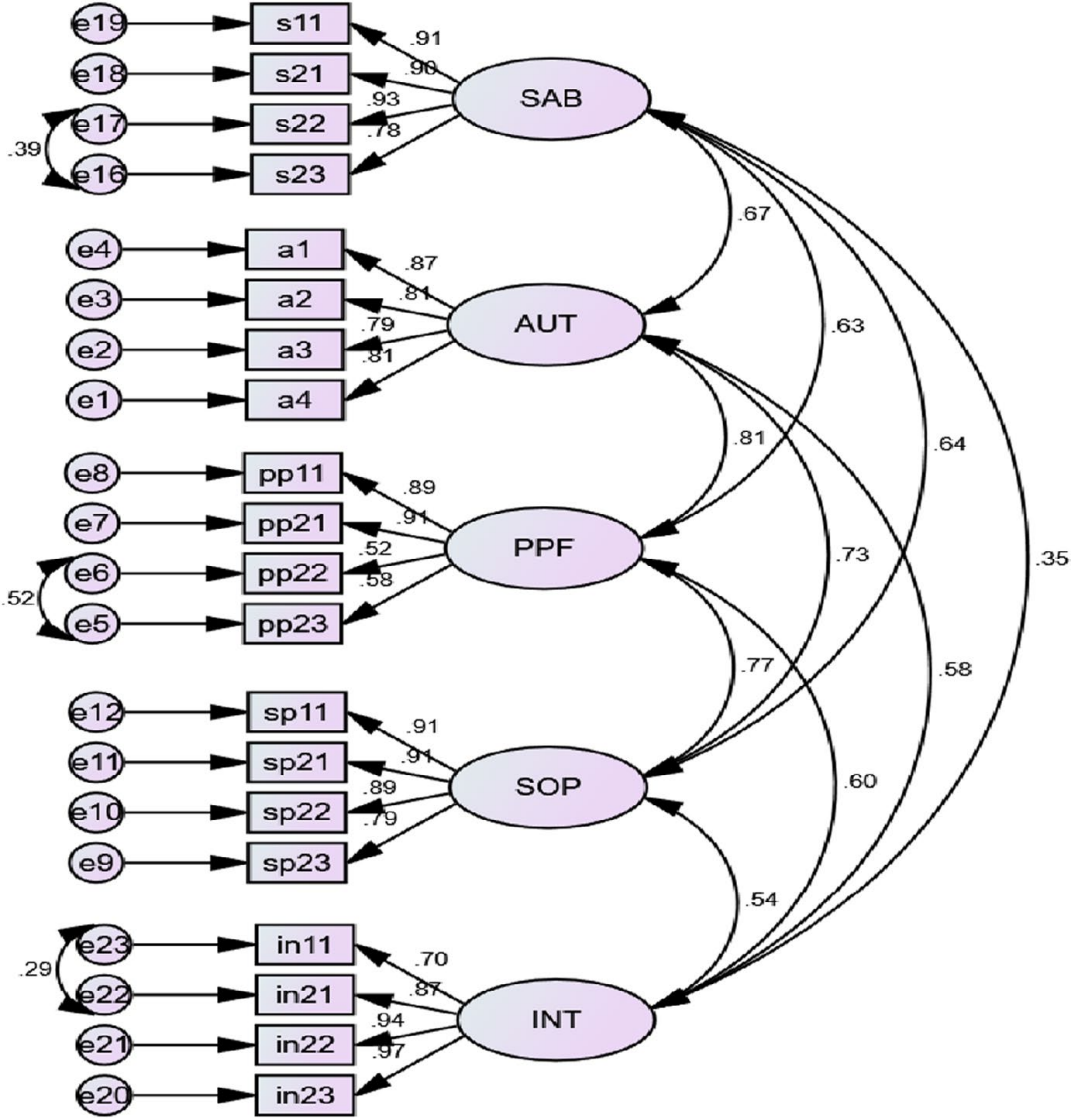
CFA of the WHOQOL‐OLD, based on data collected for the full sample. Note, SAB—Sensory Abilities (s11‐ Impairment to senses affect daily life, s21‐Rate sensory functioning, s22‐Loss of sensory abilities affect participation in activities, s23‐Problems with sensory functioning affect ability to interact), AUT—Autonomy (a1‐Freedom to make own decisions, a2‐Feel in control of your future, a3‐Able to do things you'd like to do, a4‐People around you are respectful of your freedom), PPF—Past, Present, Future (pp11‐ Happy with things to look forward to, pp21‐ Received the recognition you deserve in life, pp22‐ Satisfied with opportunities to continue achieving, pp23‐Satisfied with what you've achieved in life), SOP—Social Participation (sp11‐Satisfied with the way you use your time, sp21‐Satisfied with level of activity, sp22‐Have enough to do each day, sp23‐Satisfied with opportunities to participate in the community), INT—Intimacy (in11‐Feel a sense of companionship in life, in21‐Experience love in your life, in22‐Opportunities to love, in23‐Opportunities to be loved)

Sex specific analyses also revealed that the models in which the residuals of the aforementioned items correlate freely showed significant model improvement over the initial models. The results of sex invariance analyses based on the respecified models indicate that configural invariance (TLI = 0.93; CFI = 0.94; RMSEA = 0.06), metric invariance (ΔCFI = 0.00), and invariance of factor variance (ΔCFI = 0.00) and (ΔCFI = −0.01) have been attained. The factor structure from the CFA model and their corresponding coefficients are summarized in the Figure 1.

**TABLE 4:** Confirmatory factor analysis and sex invariance of WHO‐quality of life questionnaire

Model/Fit Indices	χ^2^(*df*)	χ^2^/*df*	TLI	CFI	RMSEA	AIC	BIC	Δχ^2^	ΔCFI
Full sample									
Original	576.98(160)***	3.61	0.92	0.94	0.09	676.98	870.31		‐
Respecified	418.73(157)***	2.67	0.95	0.96	0.06	524.73	729.66	158.25(3)***	‐
Males									
Original	320.43(160)***	2.01	0.90	0.92	0.09	420.73	557.10		
Respecified	284.86(158)***	1.80	0.92	0.93	0.08	388.86	530.68	35.57(2)***	
Females									
Original	539.33(160)***	3.37	0.90	0.92	1.00	639.33	813.36		
Respecified	424.16(157)***	2.70	0.93	0.94	0.08	530.16	714.63	115.17(1)***	
Sex Invariance									
Unconstrained	700.30(314)***	2.23	0.93	0.94	0.06	‐	‐	‐	‐
Constrained 1	715.53(329)***	2.18	0.93	0.94	0.06			15.23(15), ns	0.00
Constrained 2	730.24(342)***	2.14	0.93	0.94	0.06			14.71(13), ns	0.00
Constrained 3	813.12(367)***	2.22	0.93	0.93	0.06			82.88(25)***	−0.01

Note

Unconstrained = parameters freely estimated; Constrained 1 = factor loadings constrained; Constrained 2 = Factor variances and covariances constrained; Constrained 3 = Error variances constrained; ns = not significant.

* *p* < .001.

** *p* < .001.

*** *p* < .001.

**TABLE 5:** Confirmatory Factor Analysis and Ethnic(Regional) Invariance of the WHO‐Quality of Life Questionnaire

Model/Fit Indices	χ^2^(*df*)	χ^2^/*df*	TLI	CFI	RMSEA	AIC	BIC	Δχ^2^	ΔCFI
Full sample									
Original	576.98(160)***	3.61	0.92	0.94	0.09	676.98	870.31		‐
Respecified	418.73(157)***	2.67	0.95	0.96	0.06	524.73	729.66	158.25(3)***	‐
Accra									
Original	320.03(160)***	2.00	0.90	0.92	1.00	420.03	555.10		
Respecified	279.27(157)***	1.78	0.92	0.94	0.08	385.27	528.87	40.76(3)***	
Sunyani									
Original	304.81(160)***	1.91	0.91	0.93	0.08	404.81	545.01		
Respecified	293.52(157)***	1.87	0.91	0.93	0.08	399.52	548.13	11.29(3)***	
Navrongo									
Original	499.64(160)***	3.12	0.84	0.87	0.13	599.64	739.01		
Respecified	325.17(155)	2.09	0.92	0.93	0.09	435.17	588.48	174.47(5)***	
Regional Invariance									
Unconstrained	891.37(465)***	1.92	0.92	0.93	0.05	‐	‐	‐	‐
Constrained 1	970.28(495)***	1.96	0.92	0.93	0.05			78.91(30)***	0.00
Constrained 2	1,072.91(525)***	2.04	0.91	0.92	0.06			106.63(30)***	0.00
Constrained 3	1,428.65(575)***	2.48	0.87	0.87	0.07			355.75(50)***	−0.01

Note

Unconstrained = parameters freely estimated; Constrained 1 = factor loadings constrained; Constrained 2 = Factor variances and covariances constrained; Constrained 3 = Error variances constrained.

* *p* < .001.

** *p* < .001.

*** *p* < .001.

### 3.3 Confirmatory factor analysis and location invariance

Table 5 provides a summary of the CFA and ethnicity (regional) invariance of the WHOQOL questionnaire. Consistent with the findings in Table 4, the full sample model was respecified to achieve good model fit by allowing the error variances of the following items to correlate: Past, present, and future items #22 (Satisfied with opportunities to continue achieving) and #23 (Received the recognition you deserve in life) (MI = 92.92), Intimacy item #21 (*Feel a sense of companionship in life*) and Intimacy item #22 (*Experience love in your life*) (MI = 25.09), and sensory abilities items #22 (rate of sensory functioning) and #23 (Loss of sensory abilities affect participation in activities) (MI = 15.55). As can be seen, the resulting models were good fit to the data (TLI = 0.95; CFI = 0.96; RMSEA = 0.06). The initial models for participants from Accra and Sunyani were respecified based on the modifications above, leading to an improvement in model fit. For Accra, the fit of the respecified model is as follows: (TLI = 0.92; CFI = 0.94; RMSEA = 0.08). In addition to the above, the model for participants from Navrongo was respecified by allowing the residuals of items for Social interaction items #22 (Satisfied with level of activity) and #23 (Satisfied with opportunity to participate in community); and Autonomy items #2 (Feel in control of your future) and #4 (Able to do things you'd like) to correlate freely. The resulting model improved over the initial model and provided a good model fit to the data (TLI = 0.92; CFI = 0.93; RMSEA = 0.09). Measurement invariance analyses also revealed that configural invariance (TLI = 0.92; CFI = 0.93; RMSEA = 0.05), metric invariance (ΔCFI = 0.00), and invariance of factor variance (ΔCFI = 0.00) and error variances (ΔCFI = −0.01) have been attained.

## 4 DISCUSSION

The primary aim of this paper is to establish the factorial validity of the 24‐item WHOQOL for older adults on three selected samples from Ghana. Our overarching research question was, therefore, whether or not the WHOQOL‐OLD can be used as a reliable and valid instrument for measuring quality of life among individuals living in three geographically and linguistically different districts in Ghana. This measure has been translated into over 15 languages and within those contexts provided a good basis for the measurement of quality of life among older adults (Eser et al., 2010; Fleck et al., 2006).

In this study, we compared results of three culturally different groups using data from three translated versions of the instrument. We found reasonable internal consistency coefficients for the WHO‐QOL OLD subdimensions. The reliability coefficients associated with the six subdimensions point to a reliable instrument, showing comparable coefficients in a sample of Afrikaans‐speaking population in South Africa (Van Biljon et al., 2015).

Cross‐cultural assessment issues have been advanced over the years given the influence of culture on the assessment of psychosocial constructs. In response, we tested whether the theoretical factor structure of the WHOQOL‐OLD and the invariance of same across sex and geographical location would be similar to those reported in previous studies, notably Power and Quinn (2006). In general, the findings of the study have revealed that quality of life among adults in Ghana can be represented by the dimensions stipulated by the WHOQOL‐OLD. More importantly, the multidimensional structure of the WHOQOL‐OLD is invariant across sex and three different ethnic and linguistic groups. This finding lends support to previous studies that have used the WHOQOL‐OLD in different countries, including in South Africa (Van Biljon et al., 2015), Norway (Halvorsrud et al., 2008), Brazil (Chachamovich et al., 2008), Iran (Rezaeipandari et al., 2020), South Korea (Kim et al., 2020), and Singapore (Suárez et al., 2018). The confirmation of the structure of the modified version of the WHOQOL‐OLD in the Ghanaian samples has largely extended the multidimensional concept of quality of life beyond Western and non‐Western samples on which previous studies were validated. Although previous studies reported a six factor WHOQOL‐OLD consistent with the original scale (e.g., in Iran and South Korea), our findings support a five‐factor structure. We assume that this may be in part to translation issues or to the discussion of a subject matter that is difficult for older adults. These may be tentative assumptions that need further exploration. It is important, however, to mention that in one study, it is reported that responses about items on death and dying were ambiguous, with individuals showing fear and resignation about death (Melo et al., 2018).

Notwithstanding the sociocultural and geopolitical factors (between and within countries) that exert influence on behaviors, the study has largely re‐echoed the notion that the experience and endorsement of quality of life as a salient psychosocial construct can be similar, in accordance with the concept of universality of certain behavioral repertoires (Adjorlolo et al., 2018). Invariance across sex and geographical locations implies that performance on the WHOQOL‐OLD may not be biased (i.e., under or overestimated) by sex and geographical locations. Therefore, any mean‐level difference based on the sex of the participants and/or their geographical location on quality of life could not be attributed to the biases of the WHOQOL‐OLD for one group (Anum et al., 2019). In relation to geographical location, this finding is particularly attractive in that the WHOQOL‐OLD could be administered to samples from various regions in Ghana with essentially unique and distinct cultural practices.

A cross‐cultural measure proven to have sound psychometric properties will be useful in studying quality of life and mental health needs and to provide the opportunity for comparative analysis in Ghanaian contexts and across other LMICs.

## 5 LIMITATIONS

There are a number of limitations that need to be considered when interpreting the results from this study. Social desirability and other biases may influence participants’ response to the study measures. This possibility is heightened because self‐report measures primarily do not provide mechanisms to ascertain the accuracy of participants’ response, compared with task‐based assessment modalities. The sample size for each district selected for this study was slightly less than optimal. Estimating sample size for structural equation modeling depends on a number of factors such as number of indicators, number of factors, magnitude of factor loadings, and magnitude of factor correlations (Wolf et al., 2013) most of which were not available at the time of determining sample size. Notwithstanding the invariance of the factor structure across different geographical locations in Ghana, the generalizability of the study findings to persons in other regions not sampled for the study may be limited. Ghana is multiethnic and multilingual with at least six broad language groups (Dakubu, 2015). We studied only three of these languages, and therefore, we do not make assumptions about generalizing the findings to the rest of the country. The translation of the assessment measures to the dominant languages spoken in the selected geographical location followed the standard translation approaches recommended in previous research. In spite of this, we are aware there is the possibility of translation and administration errors that could influence participants’ response.

It should be noted that the structure of the WHOQOL‐OLD was confirmed in the Ghanaian sample following model modifications based on the modification indices. This partly raises concerns about the stability of the structure of the WHOQOL‐OLD‐R in a similar sample. The findings of this and previous studies suggest there are problems with the WHOQOL‐OLD dimension “Death and Dying” providing inadequate model fit (Chachamovich et al., 2008) and having lower correlations with other dimensions of the measure (Power et al., 2005; Van Biljon et al., 2015).

## 6 CONCLUSION

This study adds to the growing number of studies that have shown that the WHOQOL_OLD has cross‐cultural validity. However, the finding that the death and dying dimension did not measure the construct adequately provides impetus for additional studies on the underlying factor structure of the WHOQOL‐OLD in the Ghanaian population or similar populations. Given that death and dying is a major theme for the aged, future research in Ghana or on a similar population should consider reconceptualizing the items to align with Ghanaian cultural perspectives on death and dying. For example, Van der Geest, in a series of studies among rural populations, has found that Ghanaians make reference to and preference for “good death,” dying peacefully and naturally in one's old age (Van der Geest, 2004).

## CONFLICT OF INTEREST

The authors report no conflict of interest.

## CONSENT FOR PUBLICATION

All authors have given consent for publication.

## Data Availability

The data that support the findings of this study are available from the corresponding author, AA, upon reasonable request.

## References

[brb32193-bib-0001] Adjorlolo, S., Asamoah, E., & Adu‐Poku, S. (2018). Predicting delinquency by self‐reported impulsivity in adolescents in Ghana. Criminal Behaviour and Mental Health, 28(3), 270–281. 10.1002/cbm.2064 29285817

[brb32193-bib-0002] Ahmad, K. A. (2016). Global, regional, and national disability‐adjusted life‐years (DALYs) for 315 diseases and injuries and healthy life expectancy (HALE), 1990–2015: A systematic analysis for the Global Burden of Disease Study 2015. The Lancet, 388(10053), 1603–1658. 10.1016/S0140-6736(16)31460-X PMC5388857 27733283

[brb32193-bib-0003] Aikins, A. D. G. & Agyeman, C. (2017). Chronic non‐communicable diseases in low‐ and middle‐income countries: Concepts and strategies for prevention, control and advocacy. CAB Reviews: Perspectives in Agriculture, Veterinary Science, Nutrition and Natural Resources, 12(027), 1–8. 10.1079/PAVSNNR201712027

[brb32193-bib-0004] Aikins, A. D. G., & Apt, N. (2016). Ageing in Ghana: Setting priorities for research, practice and policy. Ghana Stud J., 19(1), 35–45. 10.1353/ghs.2016.0002

[brb32193-bib-0005] Anum, A., Adjorlolo, S., & Kugbey, N. (2019). Depressive symptomatology in adolescents in Ghana: Examination of psychometric properties of the Patient Health Questionnaire‐9. Journal of Affective Disorders, 256, 213–218. 10.1016/j.jad.2019.06.007 31181377

[brb32193-bib-0006] Anum, A., Washington‐Nortey, M., & Dzokoto, V. (2020). Strategic planning in LAMIC mental health research: A Ghana case study. International Journal of Mental Health, 49(2), 128–156. 10.1080/00207411.2020.1719621

[brb32193-bib-0007] Arbuckle, J. L. (2011). IBM SPSS Amos 20 User’s Guide. Amos Development Corporation. SPSS Inc.

[brb32193-bib-0008] Bonicatto, S. C., Dew, M. A., Zaratiegui, R., Lorenzo, L., & Pecina, P. (2001). Adult outpatients with depression: Worse quality of life than in other chronic medical diseases in Argentina. Social Science and Medicine, 52(6), 911–919. 10.1016/S0277-9536(00)00192-1 11234864

[brb32193-bib-0009] Chachamovich, E., Fleck, M. P., Trentini, C., & Power, M. (2008). Brazilian WHOQOL‐OLD Module version: A Rasch analysis of a new instrument. Revista de Saude Publica, 42(2), 308–316. 10.1590/S0034-89102008000200017 18372982

[brb32193-bib-0010] Cortina, J. M. (1993). What is coefficient alpha? An examination of theory and applications. Journal of Applied Psychology, 78(1), 98. 10.1037/0021-9010.78.1.98

[brb32193-bib-0011] Dakubu, M. E. K. (2015). The Languages of Ghana. Routledge.

[brb32193-bib-0012] Eser, S., Saatli, G., Eser, E., Baydur, H., & Fidaner, C. (2010). The reliability and validity of the Turkish version of the World Health Organization quality of life instrument‐older adults module (WHOQOL‐Old). Turkish Psikiyatr Dergisi, 21(1), 1. 20204903

[brb32193-bib-0013] Fleck, M. P., Chachamovich, E., & Trentini, C. (2006). Development and validation of the Portuguese version of the WHOQOL‐OLD module., 40. Revista de Saude Publica, 40, 785–791. 17301899 10.1590/s0034-89102006000600007

[brb32193-bib-0014] George, D., & Mallery, P. (2019). IBM SPSS Statistics 26 Step by Step: A Simple Guide and Reference. Routledge.

[brb32193-bib-0016] Gyasi, R. M., & Phillips, D. R. (2020). Demography, socioeconomic status and health services utilisation among older Ghanaians: Implications for health policy. Ageing International, 45, 50–71. 10.1007/s12126-018-9343-9

[brb32193-bib-0017] Halvorsrud, L., Kalfoss, M., & Diseth, Å. (2008). Reliability and validity of the Norwegian WHOQOL‐OLD module. Scandinavian Journal of Caring Sciences, 22(2), 292–305. 10.1111/j.1471-6712.2007.00523.x 18489700

[brb32193-bib-0018] Hussenoeder, F. S., Jentzsch, D., Matschinger, H., Hinz, A., Kilian, R., Riedel‐Heller, S. G., & Conrad, I. (2020). Depression and quality of life in old age: A closer look. European Journal of Ageing, 18(1), 75–83. Published online. 10.1007/s10433-020-00573-8 33746683 PMC7925788

[brb32193-bib-0019] Kim, H. Y., Nho, J. H., Kim, J. Y., & Kim, S. R. (2020). Validity and reliability of the Korean version of the world health organization quality of life instrument‐older adults module. Geriatric Nursing, 42(2), 548–554. Published Online, 10.1016/j.gerinurse.2020.10.006 33143853

[brb32193-bib-0020] Melo, R. L. P. D., Silva Júnior, E. G. D., Souto, R. Q., Leão, Í. S., & Eulálio, M. D. C. (2018). Psychometric properties of the complete version of the World Health Organization Quality of Life Assessment (WHOQOL‐OLD): Reduced response scale. Psicologia: Reflexão e Crítica, 31, 785–791. 10.1186/s41155-018-0084-1 PMC6967016 32026070

[brb32193-bib-0021] Power, M., Quinn, K., & Schmidt, S. (2005). Development of the WHOQOL‐old module. Quality of Life Research, 14, 2197–2214. 10.1007/s11136-005-7380-9 16328900

[brb32193-bib-0022] Prina, A. M., Wu, Y. T., Kralj, C., Acosta, D., Acosta, I., & Guerra, M. (2020). Dependence‐and disability‐free life expectancy across eight low‐and middle‐income countries: A 10/66 study. Journal of Aging and Health, 32(5–6), 401–409. 10.1177/0898264319825767 30698491 PMC7322974

[brb32193-bib-0023] Rezaeipandari, H., Morowatisharifabad, M. A., Mohammadpoorasl, A., & Shaghaghi, A. (2020). Cross‐cultural adaptation and psychometric validation of the World Health Organization quality of life‐old module (WHOQOL‐OLD) for Persian‐speaking populations. Health and Quality of Life Outcomes, 18(1), 1–7. 10.1186/s12955-020-01316-0 32160912 PMC7066791

[brb32193-bib-0024] Skevington, S. M., Rowland, C., Panagioti, M., Bower, P., & Krägeloh, C. (2020). Enhancing the multi‐dimensional assessment of quality of life: Introducing the WHOQOL‐Combi. Quality of Life Research, 30(3), 1–13. 10.1007/s11136-020-02661-9 33331967 PMC7952286

[brb32193-bib-0025] Suárez, L., Tay, B., & Abdullah, F. (2018). Psychometric properties of the World Health Organization WHOQOL‐BREF quality of life assessment in Singapore. Quality of Life Research, 27(11), 2945–2952. 10.1007/s11136-018-1947-8 30046975

[brb32193-bib-0026] Üstun, T. B., Chatterji, S., Mechbal, A., & Murray, C. (2005). Quality assurance in surveys: Standards, guidelines and procedures. Household sample surveys in developing and transition countries. In: United Nations Statistical Division & United Nations Department of Economic and Social Affairs (pp. 199–230). (Eds.). United Nations.

[brb32193-bib-0027] Van Biljon, L., Nel, P., & Roos, V. (2015). A partial validation of the WHOQOL‐OLD in a sample of older people in South Africa. Glob Health Action, 8(1), 28209. 10.3402/gha.v8.28209 26446286 PMC4596887

[brb32193-bib-0028] Van der Geest, S. (2004). Dying peacefully: Considering good death and bad death in Kwahu‐Tafo, Ghana. Social Science & Medicine, 58(5), 899–911. 10.1016/j.socscimed.2003.10.041 14732604

[brb32193-bib-0015] WHOQoL Group (1993). Study protocol for the World Health Organization project to develop a Quality of Life assessment instrument (WHOQOL). Quality of Life Research, 2, 153–159. 10.1007/BF00435734 8518769

[brb32193-bib-0030] WHOQol Group (1998). Development of the World Health Organization WHOQOL‐BREF quality of life assessment. Psychological medicine, 28(3), 551–558. 9626712 10.1017/s0033291798006667

[brb32193-bib-0029] Wolf, E. J., Harrington, K. M., Clark, S. L., & Miller, M. W. (2013). Sample size requirements for structural equation models: An evaluation of power, bias, and solution propriety. Educational and Psychological Measurement, 73(6), 913–934. 10.1177/0013164413495237 PMC4334479 25705052

